# Phase II study of sequential hormonal therapy with anastrozole/exemestane in advanced and metastatic breast cancer

**DOI:** 10.1038/sj.bjc.6602579

**Published:** 2005-04-26

**Authors:** R V Iaffaioli, R Formato, A Tortoriello, S Del Prete, M Caraglia, G Pappagallo, A Pisano, F Fanelli, G Ianniello, S Cigolari, C Pizza, O Marano, G Pezzella, T Pedicini, A Febbraro, P Incoronato, L Manzione, E Ferrari, N Marzano, S Quattrin, S Pisconti, G Nasti, G Giotta, G Colucci, other Goim authors

**Affiliations:** 1 INT G Pascale, Naples, Italy; 2 Med Onc Pozzuoli, Naples, Italy; 3 Frattamaggiore H, Naples, Italy; 4 Department of Oncology, PF Calvi Hospital, Noale, Venise, Italy; 5 Med Onc Taranto H, Naples, Italy; 6 Med Onc Benevento H, Naples, Italy; 7 MED ONC SA H, Naples, Italy; 8 Med Onc Nola, Naples, Italy; 9 Fatebenefratelli Benevento H, Naples, Italy; 10 Med Onc Potenza H, Naples, Italy; 11 ASL BA 1, Naples, Italy; 12 Med Onc INT Bari, Naples, Italy by the Southern Italy Oncology Group (GOIM)

**Keywords:** sequential hormonal therapy, breast cancer

## Abstract

Hormonal therapy is the preferred systemic treatment for recurrent or metastatic, post-menopausal hormone-receptor-positive breast cancer. Previous studies have shown that there is no cross-resistance between exemestane and reversible aromatase inhibitors. Exposure to hormonal therapy does not hamper later response to chemotherapy. Patients with locally advanced or metastatic, hormonal receptor positive or unknown, breast cancer were treated with oral anastrozole, until disease progression, followed by oral exemestane until new evidence of disease progression. The primary end point of the study was clinical benefit, defined as the sum of complete responses (CR), partial responses (PR) and >24 weeks stable disease (SD). In all, 100 patients were enrolled in the study. Anastrozole produced eight CR and 19 PR for an overall response rate of 27% (95% CI: 18.6–36.8%). An additional 46 patients had long-term (>24 weeks) SD for an overall clinical benefit of 73% (95% CI: 63.2–81.4). Median time to progression (TTP) was 11 months (95% CI: 10–12). A total of 50 patients were evaluated for the second-line treatment: exemestane produced one CR and three PR; 25 patients had SD which lasted ⩾6 months in 18 patients. Median TTP was 5 months. Toxicity of treatment was low. Our study confirms that treatment with sequential hormonal agents can extend the period of time during which endocrine therapy can be used, thereby deferring the decision to use chemotherapy.

Treatment of metastatic breast carcinoma is still controversial. The introduction of new cytotoxic drugs and new chemotherapic regimens has, up to now, not resulted in a significant increase in survival.

Endocrine therapy is the preferred systemic treatment for recurrent or metastatic hormone-receptor-positive post-menopausal breast cancer (Carlson and Henderson, 2003), since it is well known that breast cancers expressing the oestrogen receptor (ER) and/or progesterone receptor (PgR) are sensitive to an increasingly wide variety of hormonal therapies (Carlson, 2002). Many patients who have been treated with Tamoxifen (Tam), which is still the standard hormonal treatment for breast carcinoma in the adjuvant setting, need new drugs with antiproliferative effects on oestrogen-dependent breast tumours. The available therapies include Tamoxifen or ovarian ablation (surgical oophorectomy, radiotherapeutic ablation or pharmacologically induced) in pre-menopausal women, Tamoxifen or aromatase inhibitors (AIs) in post-menopausal women, and the progestins, androgens and high-dose oestrogens for both pre- and post-menopausal patients.

Importantly, exposure to endocrine treatment does not seem to hamper later response to chemotherapy (Taylor *et al*, 1986). Since the goal of treating metastatic breast cancer is palliative (Hortobagyi, 1998a), minimally toxic alternatives are needed to improve clinical symptoms, to maintain acceptable performance status and quality of life, and to delay the need for chemotherapy. As endocrine therapies have developed, it has become recognised that a response to one therapy is predictive of a response to further hormonal therapy (Buzdar and Hortobagyi, 1998; Hortobagyi, 1998b). The sequential use of endocrine therapy alone offers significant quality-of-life advantages over cytotoxic chemotherapy (Buzdar and Hortobagyi, 1998), since it offers disease control without the side effects associated with cytotoxic agents. The development of novel endocrine therapies may extend the period of time during which sequential endocrine therapy can be used, thereby postponing the need for cytotoxic chemotherapy. Traditionally, the sequence of endocrine therapies was determined by the relative toxicity of the respective agents, with the least toxic being used first. However, more recently, the relative efficacy of agents has become an increasingly important consideration, particularly since AIs have been shown to represent clinically relevant alternatives to Tamoxifen in women with hormone receptor-positive breast cancer (Carlson *et al*, 2003).

Anastrozole (AN), the first third-generation nonsteroidal AI approved in the USA, acts by reversibly binding to the aromatase enzyme and shows a low toxic profile, because it does not inhibit the production of adrenal steroids, nor does it determine the occurrence of thrombotic events as frequently as with tamoxifen (Bonneterre *et al*, 1999). The rationale for the use of this drug as first-line therapy in postmenopausal patients is supported by European (Robertson *et al*, 1999) and American (Nabholtz *et al*, 2000) phase III studies *vs* tamoxifen, which have demonstrated an equivalent efficacy, an increase in time to progression (TTP; 11.1 months for AN and 5.6 months for Tam) and a lower incidence of thrombotic events, indicating the possibility of considering AN as a first-line treatment for patients with advanced breast carcinoma. On the other hand, negative data have also been reported (Nabholtz *et al*, 2003).

Exemestane (Exe) is a second-generation, steroidal AI which irreversibly binds to the enzyme aromatase. The novel aromatase inactivator Exe has been evaluated extensively in phase I and II studies at dosages of up to 600 mg day^−1^. It is well tolerated and, as a consequence, a maximum-tolerated dose has not been identified (Di Salle *et al*, 1994; Johannessen *et al*, 1997). Oral Exe 10–25 mg day^−1^ suppresses plasma oestrogens to as much as 6–15% of pretreatment levels (Johannessen *et al*, 1997). In two phase II uncontrolled studies, objective response (OR) rates with Exe as second-line therapy in postmenopausal women with advanced breast cancer were 22 and 28%, and overall success rates (defined as the proportion of patients with OR or stable disease (SD) for ⩾24 weeks; sometimes described by other authors as ‘clinical benefit’ (Buzdar *et al*, 1998; Dombernowsky *et al*, 1998) were 47 and 48%, respectively (Jones *et al*, 1998; Kvinnsland *et al*, 1998).

Phase II studies suggest that there is no cross-resistance between Exe and reversible inhibitors of aromatase and that the tolerability profile is excellent. Therefore, a sequential approach with AN followed by exemestane is worth pursuing, even upon the availability of newer compounds such as fulvestrant and trilostane.

On the basis of these considerations, we performed a phase II study which evaluated the activity of AN, a type II AI, as a first-line drug, and, in the event of disease progression, of exemestane, type I, in patients with metastatic breast carcinoma.

## MATERIALS AND METHODS

### Patient selection

This study was initiated in November 2000 and closed to accrual in November 2002, was conducted at 13 sites within the Gruppo Oncologico Italia Meridionale (GOIM – Southern Italy Oncology Group). All patients were required to have a cytological or histological confirmed diagnosis of locally advanced or metastatic or primary breast cancer, not susceptible to surgical or radiological treatment and measurable disease.

All patients were required to have an ECOG performance status (WHO scale) ⩽2, and to be postmenopausal or premenopausal in treatment with LH–RH agonist. Postmenopausal women were defined as those ⩾50 years of age who had not menstruated during the preceding 12 months or who had castrate follicle-stimulating hormone levels (>40 IU l^−1^), those younger than 50 years of age who had castrate follicle-stimulating hormone levels (>40 IU l^−1^) or those who had undergone a bilateral oophorectomy.

Prior adjuvant chemotherapy or hormonal therapy with Tamoxifen for early breast cancer was permitted, provided that the patient had not received tamoxifen in the 6 months prior to entry into the trial.

Patients were required to have tumours that were ER-positive and/or PgR-positive or were of unknown receptor status. Patients with negative receptors or with one negative and the other unknown were excluded from the study. Other exclusion criteria were patients with cerebral metastasis, diffuse bilateral lymphangitis or patients with only osteoblastic bone metastasis, or with both lytic and osteoblastic lesions in which fewer than 50% of the lesions were lytic, when there were not other measurable lesions.

Patients with more than one previous chemotherapeutic line for metastatic disease, prior therapy with AIs, uncontrolled cardiologic dysfunctions, a low compliance to treatment, and other concomitant or previous malignancies (except for basal cell carcinoma and carcinoma *in situ* of the cervix) were excluded, too. The study was approved by the Ethic Committees of the participating centres and all patients gave their informed written consent.

### Treatment plan

Eligible patients were treated as follows: AN, one 1 mg tablet daily, until clear evidence of progression; exemestane, one 25 mg tablet daily, from evidence of progression after AN therapy until further evidence of disease progression. Subsequent therapy was left up to the discretion of the investigator, and follow-up was performed until death.

### Patient evaluations

Baseline screening assessments were completed 2 weeks before the treatment. These assessments included demographic information, a complete history and a clinical visit to document the sites of disease. Laboratory studies included chest X-ray, liver scan by ultrasound, computed tomography (CT) scan, or magnetic resonance imaging, bone scan, and bone survey or X-rays of areas that were found suggestive of abnormality on the bone scan. Blood samples were collected for haematology and blood chemistry (general and specific, tumour markers). A history of disease-related symptoms was also documented.

A clinical visit, general and specific (tumour markers) laboratory procedures, total-body CT scan were repeated at a 3-months interval; bone scans were repeated every 12 months.

Response to treatment was evaluated according to RECIST criteria. Time to progression was measured in all patients as the time (in months) from the start of study drug to the date of evidence of progressive disease or death (or last follow-up in absence of unfavourable event). Toxicity was evaluated according to CTC criteria (CTC version 2.0. Cancer Therapy Evaluation Program. Common Toxicity Criteria, Version 2.0. DCTD, NCI, NIH, DHHS. March 1998. Publish Date: April 1999).

Patients were withdrawn from active treatment if there was evidence of clinically significant breast cancer progression, or a serious adverse event. Patient noncompliance with protocol procedures, or unwillingness or inability to continue the trial was also a reason for patient removal from the trial.

### Statistical considerations

The primary objective of the study was to evaluate the study drugs in terms of clinical benefit (complete response (CR)+partial response (PR)+stabilisation of disease (SD) >6 months). Secondary objectives were major (CR+PR) response rate, TTP and toxicity. The sample size calculation for both single-stage studies was performed as proposed by A'Hern (2001), this method being an exact version of the algorithm first presented by Felming (1982).

The AN evaluation required 93 subjects to decide whether the proportion of patients with a clinical benefit (*P*) was ⩽50% or ⩾65%. If the number of patients with clinical benefit was ⩾55, the hypothesis that *P*⩽50% was rejected with a target error rate of 0.050 and an actual error rate of 0.048. If the number of patients with clinical benefit was ⩽54, the hypothesis that *P*⩾65% was rejected with a target error rate of 0.100 and an actual error rate of 0.099.

The exemestane evaluation required 47 subjects to decide whether *P* was ⩽20% or ⩾40%. If the number of patients with clinical benefit was ⩾15, the hypothesis that *P*⩽20% was rejected with a target error rate of 0.050 and an actual error rate of 0.037. If the number of patients with clinical benefit was ⩽14, the hypothesis that *P*⩾40% was rejected with a target error rate of 0.100 and an actual error rate of 0.099.

## RESULTS

### Patient population

Between November 2000 and November 2002, 100 patients aged 30–93 years (median: 66) were enrolled in the study. The patient characteristics are presented in Table 1.

### First-line AN

Eight CRs (8%) and 19 PRs (19%) were observed, providing an OR rate of 27% (95% CI: 18.6–36.8%). In all, 46 additional patients had a long-term disease stabilisation (SD after ⩾6 months), providing a clinical benefit rate of 73% (95% CI: 63.2–81.4); thus, AN was considered as active at the level of clinical interest as above defined. Five other patients had a stabilisation lasting 4–6 months, and 19 had a PD (see Table 2). Three patients were not evaluable for response: one had a severe osteo-arthromyalgia, and two left the study by their own choice (in the absence of PD or any evident adverse reaction).

**Figure 1:**
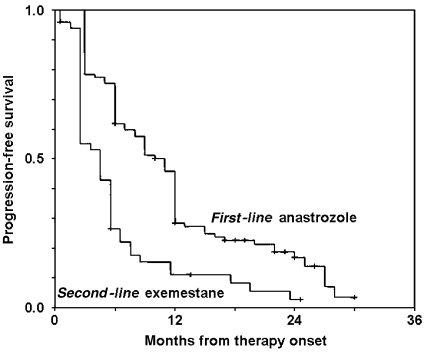
Progression-free survival.

In all, 84 patients discontinued the first-line AN therapy, three before evaluation of activity (see above) and 81 because of a PD. The median duration of AN therapy was 9 months (range: 3–30+ months), for a median follow-up of 15 months (range: 3–32+ months). The median Kaplan–Meier progression-free survival estimate was 11 (95% CI: 10–12) months (Figure 1).

First-line AN was well tolerated. No patient manifested any thrombo-embolic event or bone fracture. Seven (7%) patients had a grade 1–2 osteo-articular pain, 20 (20%) manifested hot flushes and 19 (19%) manifested a grade 1–2 nausea and vomiting.

### Second-line exemestane

Five of 13 treatment centres did not agree to participate in the activity trial of exemestane in patients progressing after first-line AN; thus, 50 patients were eligible for this study. One CR (2%) and three PR (6%) were observed in patients treated with exemestane, for an overall response rate of 8% (95% CI: 2.2–19.2%) (see Table 2). The patient who achieved a CR had had a CR also following first-line AN therapy; on the other hand, all of the three patients who achieved a PR following exemestane had progressed while on previous AN therapy.

In all, 18 additional patients had a long-term disease stabilisation (⩾6 months), providing a clinical benefit rate of 44% (95% CI: 30.0–58.7%); seven other patients had a stabilisation lasting 4–6 months. Among the 25 patients who had SD following exemestane therapy, 24 had previously responded to AN; however, the only patient with SD who had previously had a PD following AN had the longest SD duration (25+ months).

Altogether, 20 patients had a PD at exemestane evaluation, and one patient was not evaluable for response because of a treatment-limiting gastro-intestinal pain.

In all, 46 patients discontinued the second-line, exemestane therapy; 45 because of a PD, and one because of gastro-intestinal pain. The median duration of exemestane therapy and follow-up was 5 months (range: 3–25+ months). The median Kaplan–Meier, progression-free survival estimate was 5 (95% CI: 3–7) months (Figure 1).

Also, second-line exemestane was well tolerated. No patient manifested any thrombo-embolic event or bone fracture. Five (10%) patients had a grade 1–2 osteo-articular pain, 10 (20%) manifested hot flushes and 12 (24%) manifested a grade 1–2 nausea and vomiting. Moderate grade fatigue was reported in 14% of patients.

## DISCUSSION

Over the last few years, with the widespread availability of letrozole, AN and exemestane, the goal of developing highly effective, well-tolerated, orally active AIs has been realised.

Data from two recent trials that compared AN with megestrol Acetate in second line confirmed that AN was well tolerated, produced response rates similar to megestrol and determined a significant improvement in global survival although it was not associated with the increase in weight produced by megestrol acetate (Buzdar *et al*, 1996).

Two similar studies with letrozole in patients in relapse with adjuvant tamoxifen or first-line antioestrogens, demonstrated that letrozole can obtain efficiency results similar or superior to megestrol acetate (Dombernowsky *et al*, 1998; Buzdar *et al*, 2001).

Exemestane, the most recently introduced AI, obtained a significant improvement in survival compared to megestrol acetate (Kaufmann *et al*, 2000).

Both AN and letrozole seem to offer greater effectiveness than tamoxifen in the first-line treatment of women in menopause and with positive receptors (Bonneterre *et al*, 2001; Mouridsen *et al*, 2001).

Now there is clear evidence in the international scientific community that these third-generation AIs represent credible therapeutic options such that they can be used in first line at the first relapse in post-menopausal women whether or not they have received adjuvant treatment with tamoxifen. The issue of sequential hormonal treatment has been addressed in clinical studies in which AN was given either before tamoxifen or upon progression on tamoxifen (Thürlimann *et al*, 2000, 2002). Encouraging overall response rates and meaningful clinical benefit were observed in all of these studies.

Exemestane showed activity in patients in progression after prior treatment with nonsteroidal AIs. Good results were observed in terms of OR and clinical benefit in a comprehensive percentage of over 30% (Lønning *et al*, 2000). In so much as these data are less favourable than those reported with tamoxifen after AN, it is, in any case, confirmed that there is an interest in using exemestane in third line for patients pretreated both with tamoxifen and with non-steroidal AIs.

The rationale for the sequential use of triazole derivatives, such as AN, and steroidal compounds, such as exemestane, lies in the difference between the two classes of compounds with respect to their biochemical action on the aromatase enzyme. In fact, while nonsteroidal compounds bind to the p450 site of the aromatase complex, the steroidal compounds bind to the substrate-binding pocket.

The results of our work show several interesting aspects. First of all, the toxicity level was especially low; treatment was suspended in only two cases, once in first-line therapy and once in second line, and there were no noteworthy signs of thromboembolism. This allowed sequential treatment to be carried out even for long periods of time with a good quality of life and absolute normalcy of vital functions in almost all patients. If the total of ORs (CR+PR) is consistent with that described in international literature for first-line treatments, the duration of response and TTP was considerable. Clinical benefit was even more important because of the high percentage of long-lasting SD. This very encouraging result can probably be explained by the high percentage of patients with a known receptorial state and the low percentage of visceral metastases.

Results of second-line responses showed clear evidence of an absence of cross-resistance between the two treatments, which is the consequence of the different mechanism of action. The observed high TTP in second line is also to be noted. There is no doubt that, overall, the data of our study confirm the usefulness of sequential hormonal treatment for patients with adequate clinical indication. A brief reflection should be made on the drop-out of about 50% of the patients in second-line treatment. This is frequently due to a widely shared opinion that chemotherapy could be a better therapeutic option in this setting. This view is very common especially among patients and relatives, and does of course influence medical behaviour.

In conclusion, our study gives clear insights into the feasibility and activity of a sequential hormonal approach in the treatment of selected patients with advanced breast cancer. Future studies with this sequential combination of AN and exemestane will help to clarify the real allocation in the therapeutic armamentarium against advanced breast cancer. Along with the use of new upcoming hormonal agents, this may represent a valuable approach which can, at least, defer the start of chemotherapy.

## Figures and Tables

**Table 1:** Demographic characteristics

			***n***	**%**
**Characteristic (100 patients)**	***n***	**%**	**Pt treated with exemestane (50 patients)**	
*Age (years)*				
Median	66			
Range	30–93			
				
*Prior adjuvant treatment*				
Hormonal only	21	21		
Cytotoxic only	25	25		
Both	30	30		
None	15	15		
Unknown	9	9		
				
*Sites of metastatic disease*				
Bone	52	52	30	60
Skin	11	11	6	12
Lymph	16	16	9	18
Pleural effusion	6	6	5	10
Liver	19	19	15	30
Lung	14	14	10	20
Breast	17	17	10	20
				
*Numbers of sites involved*				
1	64	64	23	46
2	26	26	15	30
3	8	8	10	20
>4	2	2	2	4
				
*Performance Status*				
0	43	43	21	42
1	40	40	19	38
2	17	17	10	20
				
*Receptor status*				
ER+, PgR+	66	67		
ER+, PgR−	8	8		
ER+, PgR unknown	0	0		
ER−, PgR+	5	5		
ER unknown, PgR+	0	0		
Unknown	19	19		

**Table 2:** Objective tumour response rates

	**Anastrazole 1 mg**	**Exemestane 25 mg**
**Response**	***n*=100 (%)**	***n*=50 (%)**
CR	8 (8%)	1 (2%)
PR	19 (19%)	3 (6%)
Objective response rate	27 (27%)	4 (8%)
SD⩾24 weeks	46 (46%)	18 (36%)
Clinical benefit (CR+PR+SD⩾24 weeks)	73 (73%)	22 (44%)
SD<24 weeks	5 (5%)	7 (14%)
Progression	19 (19%)	20 (40%)
