# Phase II study of sequential hormonal therapy with anastrozole/exemestane in advanced and metastatic breast cancer

**DOI:** 10.1038/sj.bjc.6602756

**Published:** 2005-09-05

**Authors:** R V Iaffaioli, R Formato, A Tortoriello, S Del Prete, M Caraglia, G Pappagallo, A Pisano, F Fanelli, G Ianniello, S Cigolari, C Pizza, O Marano, G Pezzella, T Pedicini, A Febbraro, P Incoronato, L Manzione, E Ferrari, N Marzano, S Quattrin, S Pisconti, G Nasti, G Giotta, G Colucci, and other Goim authors

RV Iaffaioli^1^, R Formato^1^, A Tortoriello^2^, S Del Prete^3^, M Caraglia^3^, G Pappagallo^4^, A Pisano^2^, F Fanelli^5^, G Ianniello^6^, S Cigolari^7^, C Pizza^8^, O Marano^8^, G Pezzella^5^, T Pedicini^9^, A Febbraro^9^, P Incoronato^2^, L Manzione^10^, E Ferrari^1^, N Marzano^11^, S Quattrin^2^, S Pisconti^5^, G Nasti^1^, G Giotta^12^, G Colucci^12^, and other Goim authors

**Correction to:** *British Journal of Cancer* (2004) **92**, 24–29. doi:10.1038/sj.bjc.6602579

Due to an author error, the contributing author list was incorrect. The correct list is given below:

RV Iaffaioli, R Formato, A Tortoriello, S Del Prete, M Caraglia, G Pappagallo, A Pisano, V Gebbia, F Fanelli, G Ianniello, S Cigolari, C Pizza, O Marano, G Pezzella, T Pedicini, A Febbraro, P Incoronato, L Manzione, E Ferrari, N Marzano, S Quattrin, S Pisconti, G Nasti, G Giotta, G Colucci and other Goim authors

